# Exceptional Finding of Double Pannus in Aortic and Mitral Mechanical Prosthetic Valves Using Noninvasive Imaging: A Case Report

**DOI:** 10.7759/cureus.114917

**Published:** 2026-08-21

**Authors:** Meriem Boumaaz, Amal Hsain, Chaimaa Abou Eddahab, Younes Moutakiallah, Zouhair Lakhal, Iliyasse Asfalou, Aatif Benyass

**Affiliations:** 1 Department of Cardiology, Mohammed V Military Teaching Hospital, Mohammed V University, Rabat, MAR; 2 Cardiology Center, Mohammed V Military Teaching Hospital, Mohammed V University, Rabat, MAR; 3 Department of Cardiology, Mohammed V University, Rabat, MAR; 4 Department of Cardiac Surgery, Mohammed V Military Teaching Hospital, Rabat, MAR; 5 Department of Interventional Cardiology, Mohammed V Military Teaching Hospital, Rabat, MAR

**Keywords:** cardiac computed tomography, mechanical prosthetic valves, obstruction, pannus, transesophageal echocardiography

## Abstract

Pannus formation is a rare but clinically important complication of mechanical prosthetic valves. The incidence of pannus in aortic prostheses is much higher than that in mitral prostheses, the latter being more prone to thrombosis. However, the presence of pannus in both prostheses is an exceptional finding. The authors present the case of a 61-year-old woman with a long history of rheumatic multivalvular disease. She had undergone three cardiac surgeries for mechanical valve prosthesis replacement in the aortic, mitral, and tricuspid positions. She was hospitalized for atypical angina and blockpnea. Transthoracic echocardiography revealed dysfunction of both the aortic and mitral mechanical prostheses due to a subvalvular mass. Transesophageal echocardiography (TEE) and CT scan showed interference from this mass, which had the appearance of pannus; this was confirmed intraoperatively, and the pannus was removed. The postoperative course was favorable. This case reports a rare clinical situation and underlines the significant role of cardiac CT and TEE in the assessment of subvalvular pannus formation in mechanical valves.

## Introduction

Pannus formation, which is defined as fibroconnective tissue ingrowth from the sewing ring of a prosthetic valve, is uncommon, but prosthetic valve obstruction by pannus can be serious [1]. Prosthetic mechanical valve function is traditionally evaluated using transthoracic echocardiography (TTE) and cinefluoroscopy. However, assessment by a single TTE examination may be limited because of suboptimal visualization of valve motion in the presence of transprosthetic pressure gradients [2]. Moreover, identification of the exact cause of prosthetic valve dysfunction, such as thrombus or pannus, by echocardiography or cinefluoroscopy can sometimes be difficult [3]. Recent technological refinements have made this difficult diagnosis possible using ECG-gated CT, which provides adequate images in 90% of cases and can differentiate pannus from fresh and organized thrombus [4].

## Case presentation

Patient information

A 61-year-old woman with a history of recurrent childhood tonsillitis requiring tonsillectomy and inadequately treated rheumatic fever subsequently developed rheumatic multivalvular heart disease associated with chronic atrial fibrillation. In 1996, she underwent double mechanical valve replacement with a 29-mm St. Jude mitral prosthesis and a 19-mm St. Jude aortic prosthesis, together with tricuspid commissurotomy. Owing to progressive tricuspid stenosis, she required a third valve intervention in 2019, consisting of mechanical tricuspid valve replacement with a 29-mm ATS prosthesis. The patient had remained compliant with long-term anticoagulation therapy.

Five years after her last surgery, she presented to the emergency department with nonradiating chest pain associated with orthopnea and progressive exertional fatigue that had gradually worsened over the preceding three years.

Clinical findings

On admission, she was hemodynamically stable, with a temperature of 37 °C, a heart rate of 77 beats/min, a blood pressure of 112/63 mmHg, a respiratory rate of 17 breaths/min, and an oxygen saturation of 98% on room air. Cardiac auscultation revealed diminished opening and closing sounds of the aortic mechanical prosthesis without any audible cardiac murmur. Pulmonary examination was unremarkable, with no crackles or signs of pulmonary congestion. There was no peripheral edema, jugular venous distension, or other signs of overt heart failure. The remainder of the physical examination was unremarkable.

Diagnostic assessment

**Figure 1:**
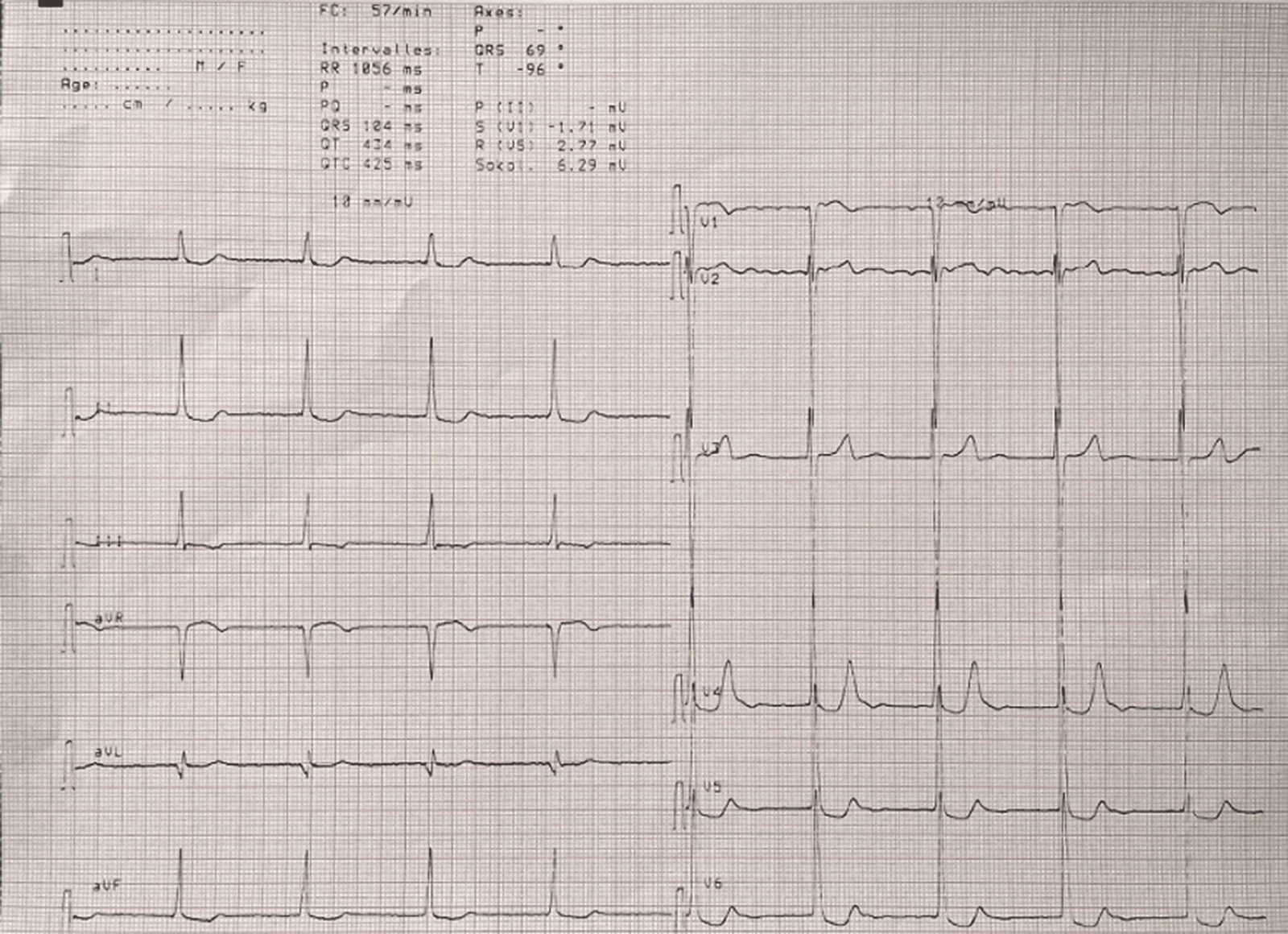
ECG showing atrial fibrillation with a ventricular rate of 57 beats/min, normal electrical axis, and left ventricular hypertrophy with a strain pattern.

ECG showed atrial fibrillation with a ventricular rate of 57 beats/min, normal electrical axis, and left ventricular hypertrophy with a strain pattern (Figure 1).

**Figure 2:**
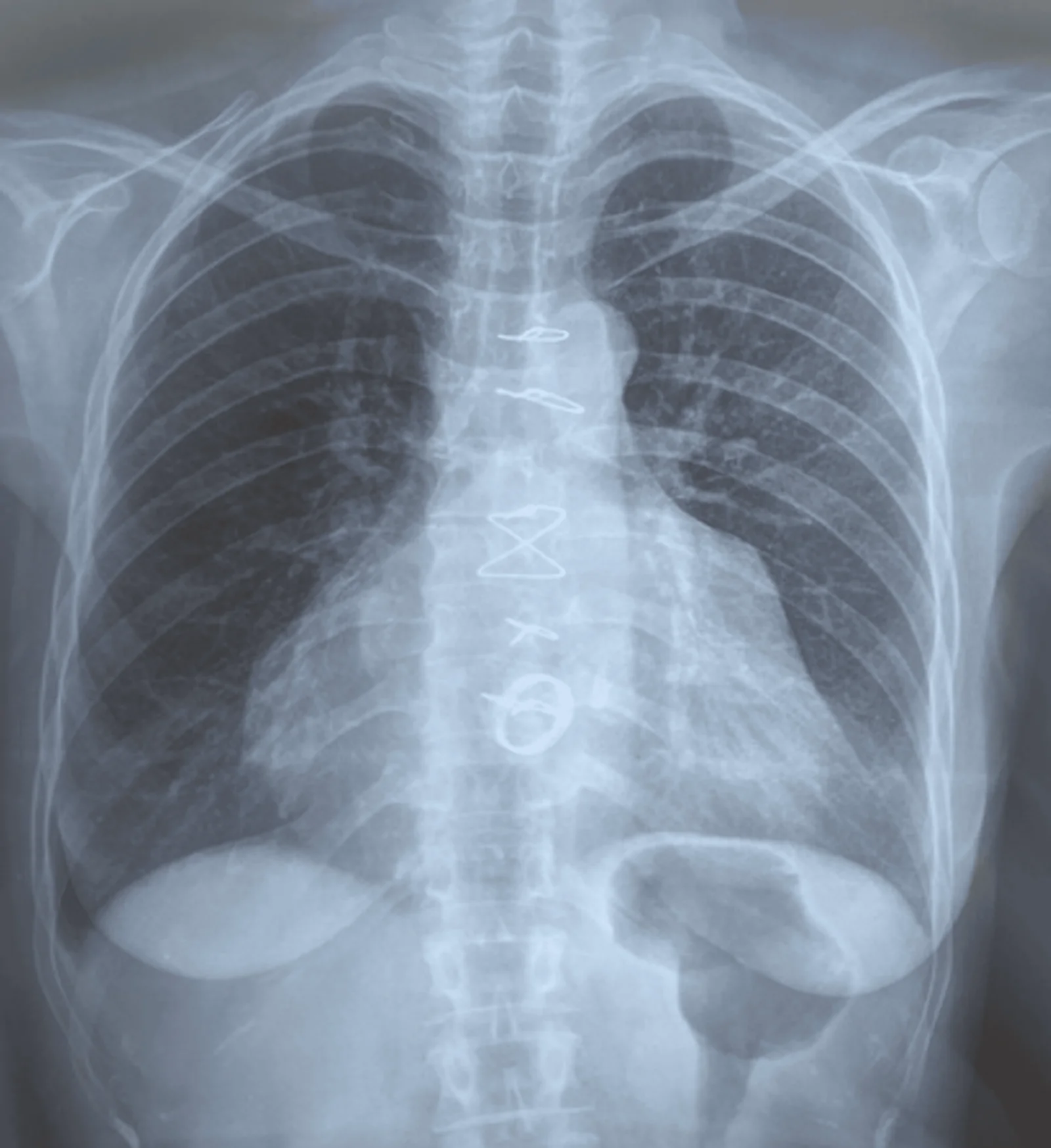
Standard chest X-ray showing cardiomegaly predominantly due to left ventricular and left atrial enlargement, with visualization of the mechanical tricuspid prosthesis and median sternotomy wires from previous cardiac surgeries.

Chest X-ray revealed enlargement of the left ventricle and left atrium, with visualization of the tricuspid prosthetic valve (Figure 2).

Laboratory investigations revealed no evidence of active infection. Complete blood count, coagulation profile, and renal and liver function tests were within normal limits. The international normalized ratio was 3.5, consistent with adequate anticoagulation.

**Figure 3:**
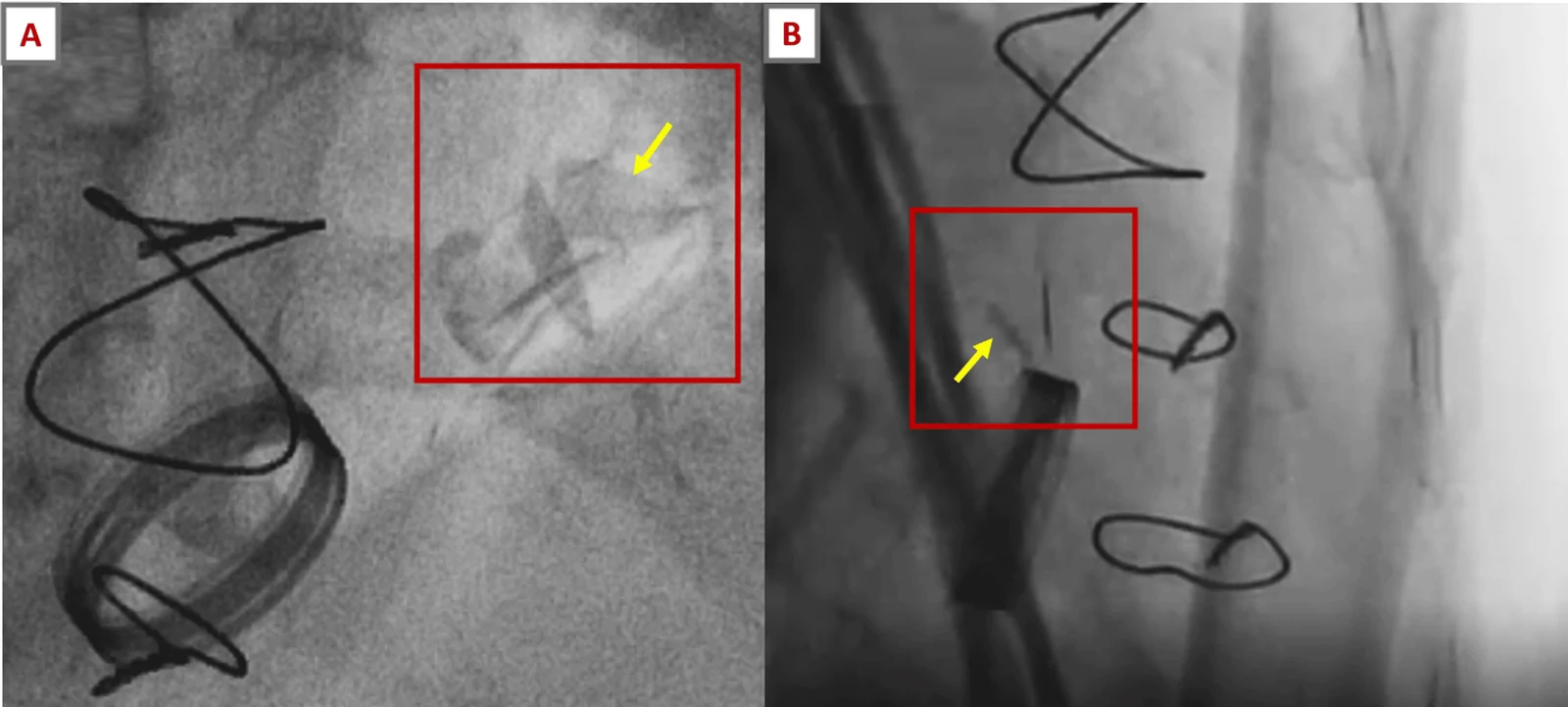
Fluoroscopic images of the mitral and aortic prosthetic valves. (A) The yellow arrow indicates the restricted leaflet, resulting in incomplete opening of the mechanical mitral valve. (B) The yellow arrow indicates the restricted leaflet, resulting in incomplete opening of the mechanical aortic valve.

Cinefluoroscopy was performed and demonstrated preserved motion of one leaflet of both the mitral and aortic prosthetic valves, whereas the second leaflet of each prosthesis was nearly completely immobilized. The tricuspid tilting-disc prosthesis showed normal mobility (Figure 3).

**Figure 4:**
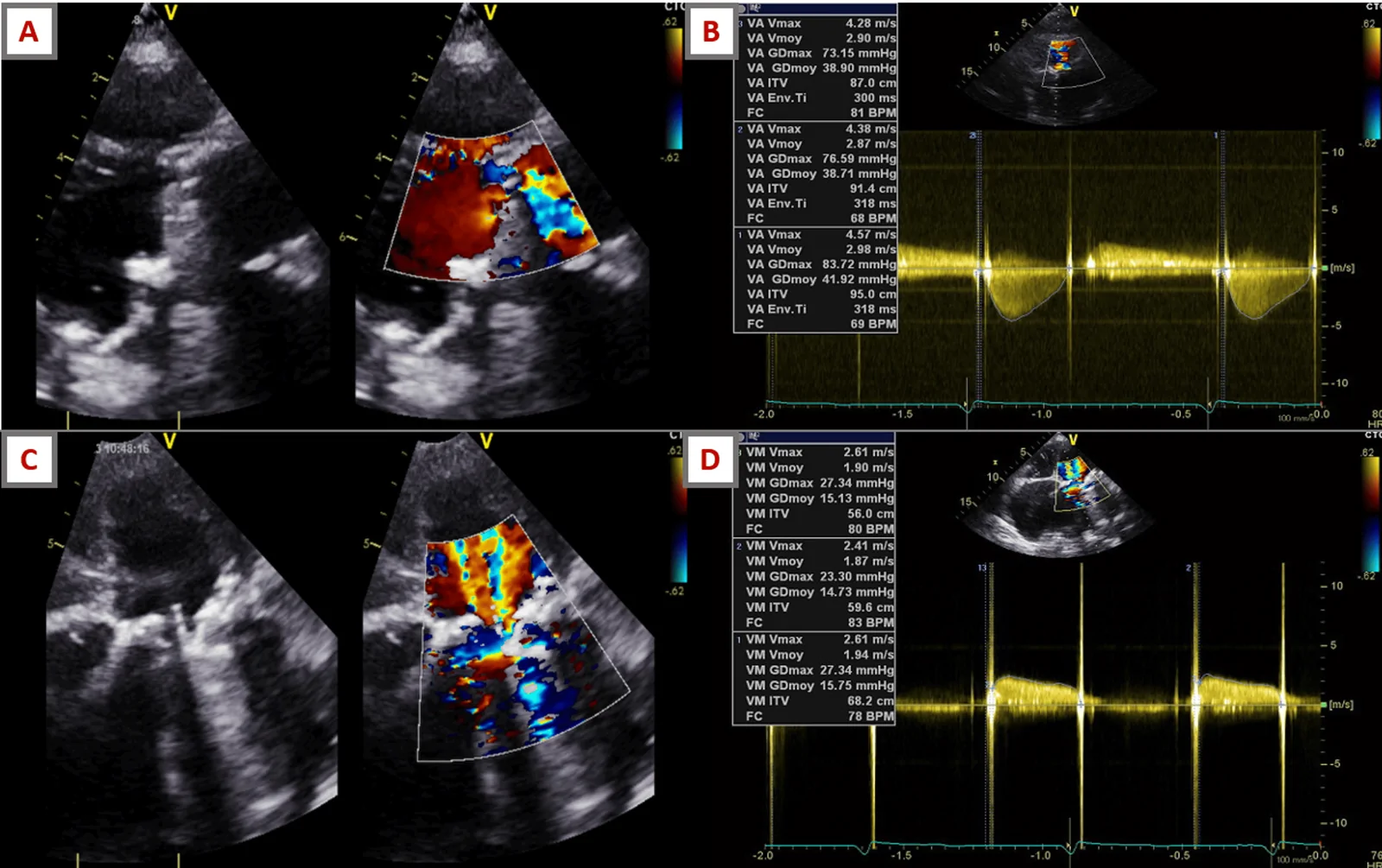
TTE images showing markedly elevated transprosthetic gradients across both the mitral and aortic prostheses. (A) Color Doppler imaging demonstrating flow acceleration with aliasing at the level of the aortic prosthesis. (B) Continuous-wave Doppler across the aortic prosthetic valve showing a markedly elevated mean gradient of 40 mmHg averaged over multiple cardiac cycles. (C) Color Doppler imaging demonstrating significant flow acceleration with aliasing at the level of the mitral prosthesis. (D) Continuous-wave Doppler across the mitral prosthetic valve showing a markedly elevated mean gradient of 15.2 mmHg averaged over multiple cardiac cycles. TTE, transthoracic echocardiography

TTE was subsequently performed to confirm the fluoroscopic findings and assess the hemodynamic consequences of the prosthetic valve dysfunction. It revealed a nondilated left ventricle with preserved systolic function and markedly elevated transprosthetic gradients across both the aortic and mitral mechanical prostheses, with mean gradients of 40 mmHg and 15.2 mmHg, respectively. The tricuspid prosthesis remained functional without evidence of significant dysfunction (Figure 4).

To further investigate the cause of the elevated transprosthetic gradients and restricted leaflet motion, transesophageal echocardiography (TEE) was performed to characterize the obstructive lesion and differentiate pannus formation from prosthetic valve thrombosis.

**Figure 5:**
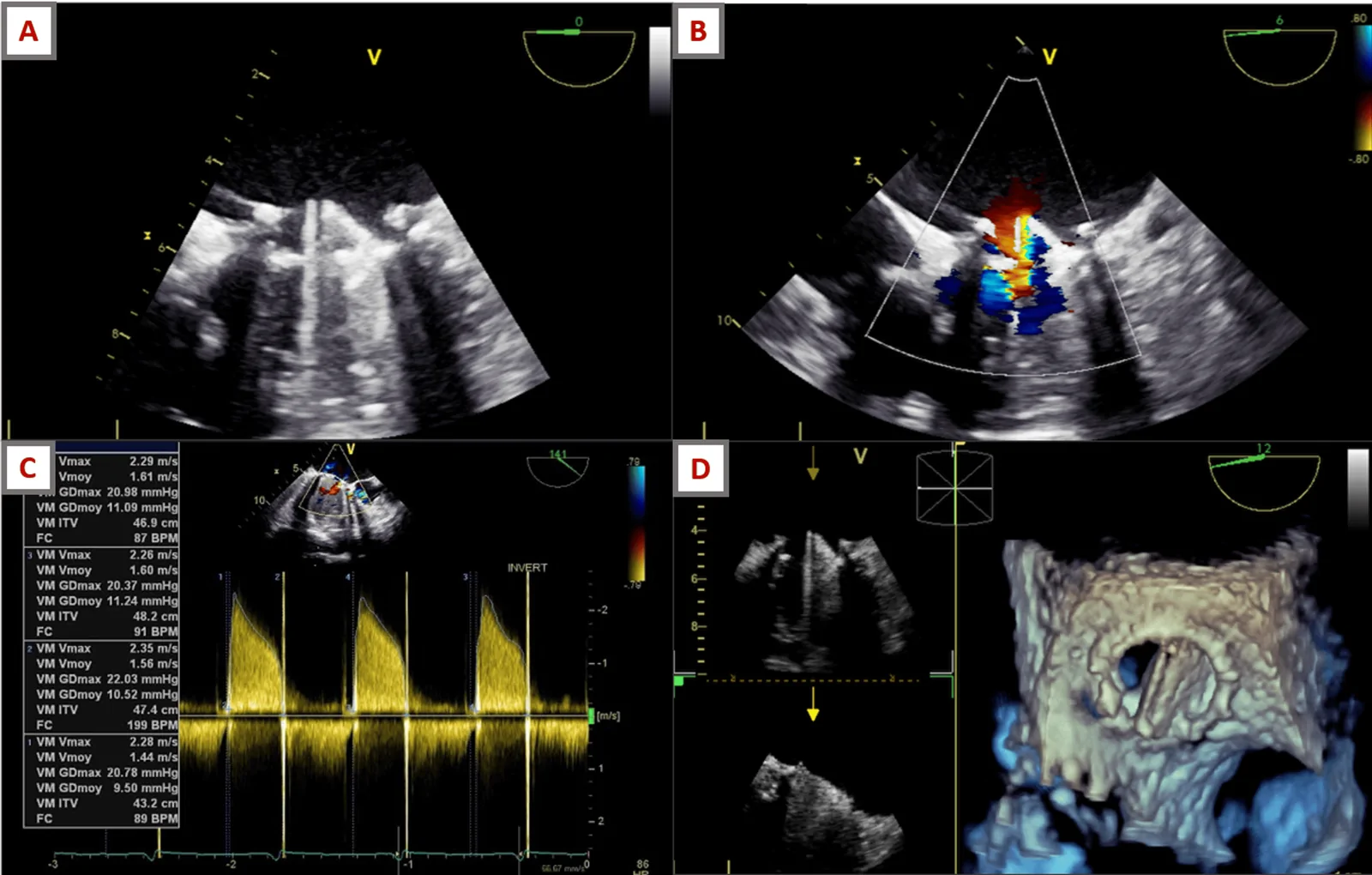
TEE images showing obstruction of one leaflet of the mechanical mitral prosthesis caused by giant pannus formation. (A) Two-dimensional TEE image demonstrating immobilization of one leaflet of the mechanical mitral valve. (B) Color Doppler imaging showing absence of blood flow through the obstructed leaflet orifice. (C) Continuous-wave Doppler demonstrating an elevated mean transmitral gradient of 11 mmHg averaged over multiple cardiac cycles. (D) Three-dimensional reconstruction illustrating leaflet obstruction caused by giant pannus formation. TEE, transesophageal echocardiography

TEE demonstrated obstruction of one leaflet of the mitral prosthesis caused by giant pannus formation, resulting in significant prosthetic stenosis with a mean transmitral gradient of 11 mmHg (Figure 5).

**Figure 6:**
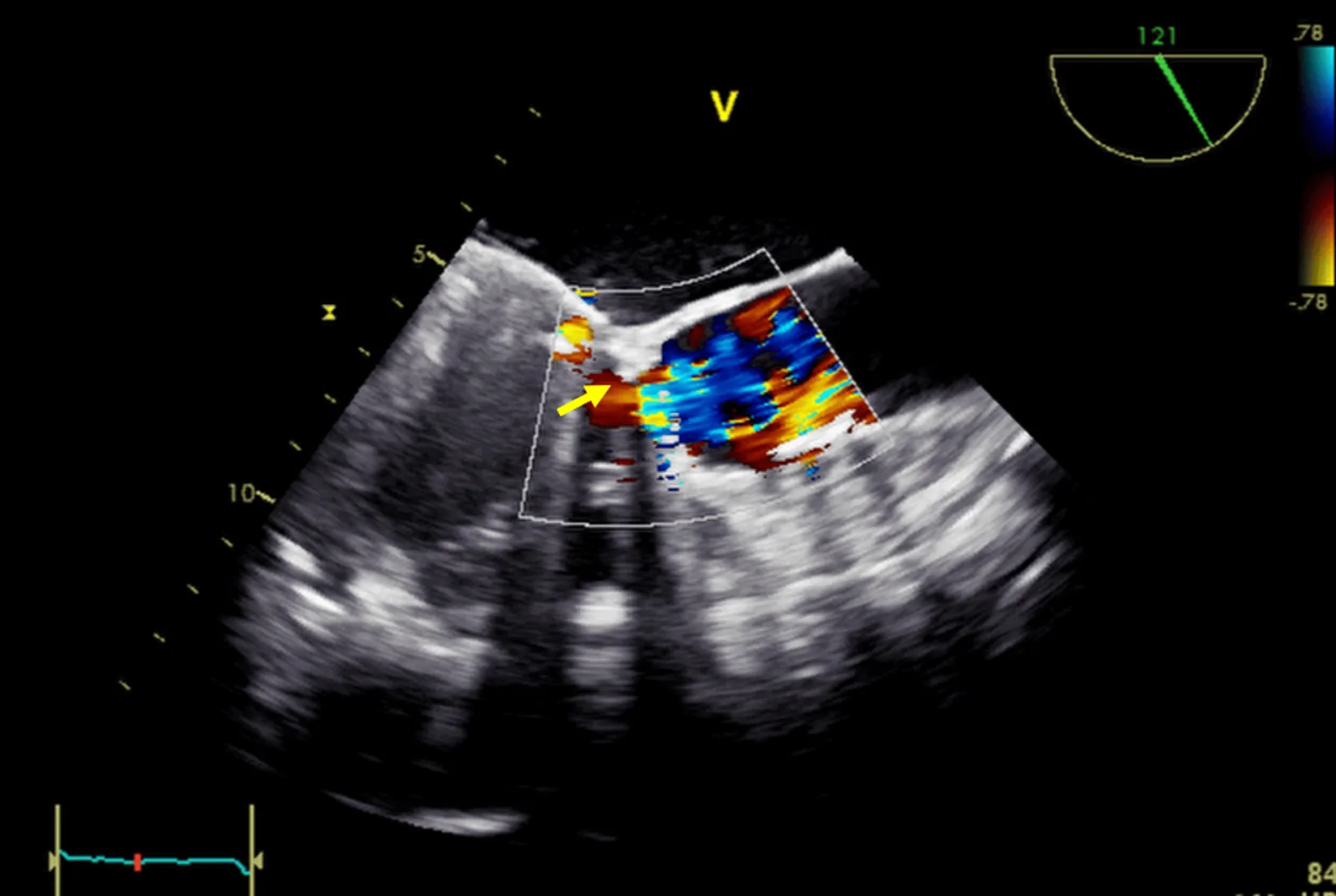
TTE imaging showing a pannus-like mass on the ventricular side of the aortic prosthesis (yellow arrow), associated with marked flow acceleration on color Doppler imaging. TTE, transthoracic echocardiography

In addition, a small pannus-like mass was identified on the ventricular side of the aortic prosthesis and was associated with immobilization of one leaflet. No thrombus was detected in the left atrial appendage (Figure 6).

**Figure 7:**
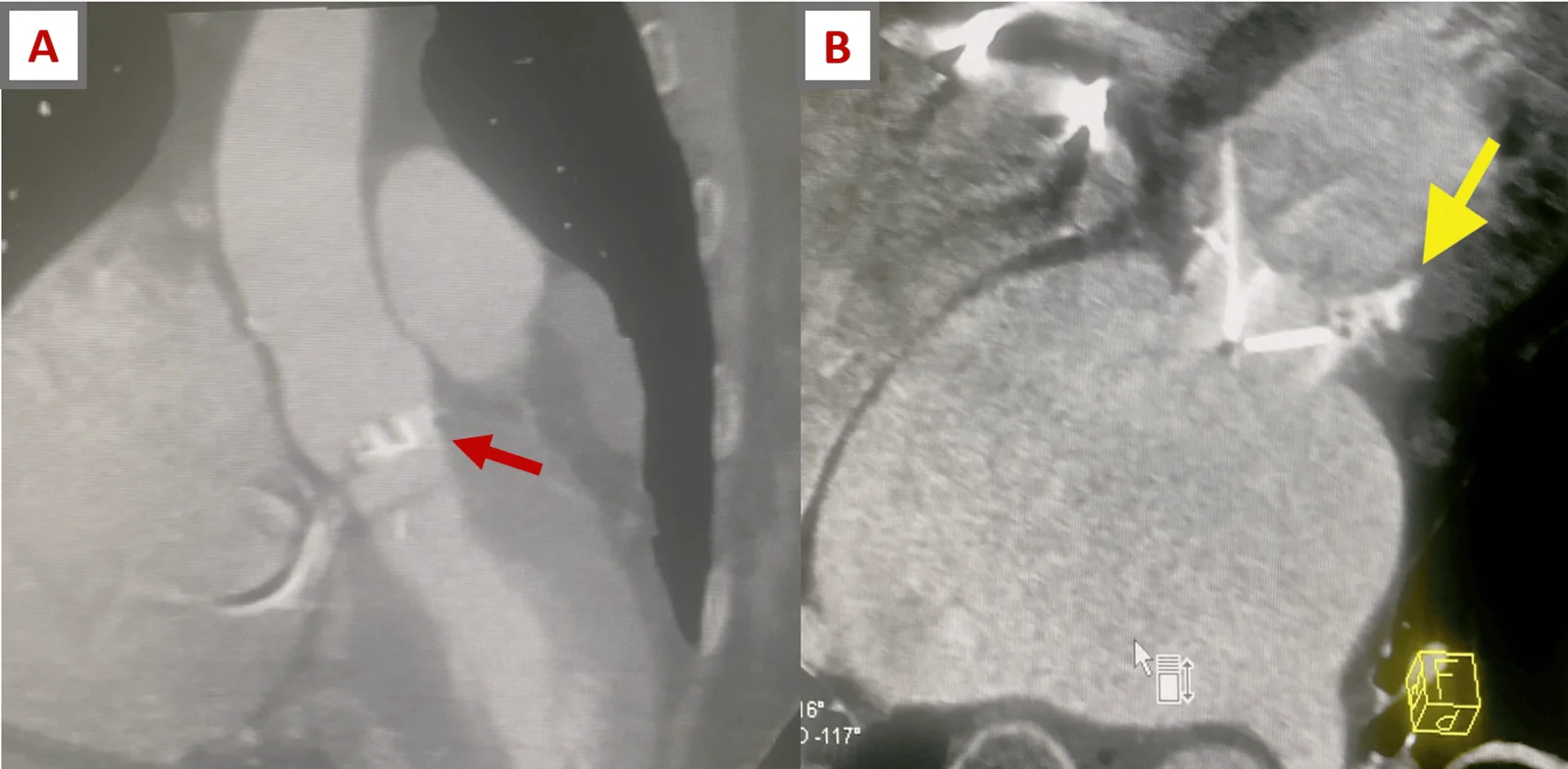
Cardiac CT of the mechanical valves in the mitral and aortic positions. (A) The red arrow indicates a dense subvalvular pannus located on the ventricular side of the mechanical aortic prosthesis. The lesion appears as a focal fibrous tissue overgrowth extending from the prosthetic ring toward the leaflet, resulting in restricted leaflet motion and prosthetic valve obstruction. (B) The yellow arrow indicates a dense pannus formation adjacent to the lateral aspect of the mitral prosthetic disc. The fibrous tissue extends toward the leaflet and contributes to impaired disc mobility and prosthetic valve obstruction.

To further characterize the periprosthetic lesions and confirm the diagnosis, cardiac CT was performed. CT demonstrated subvalvular pannus formation involving both the mitral and aortic prostheses, resulting in leaflet restriction and prosthetic valve obstruction (Figure 7).

Diagnosis

Concurrent obstructive pannus formation involving both the mechanical mitral and aortic prosthetic valves, causing severe prosthetic valve dysfunction with leaflet immobilization and elevated transprosthetic gradients.

Therapeutic intervention

**Figure 8:**
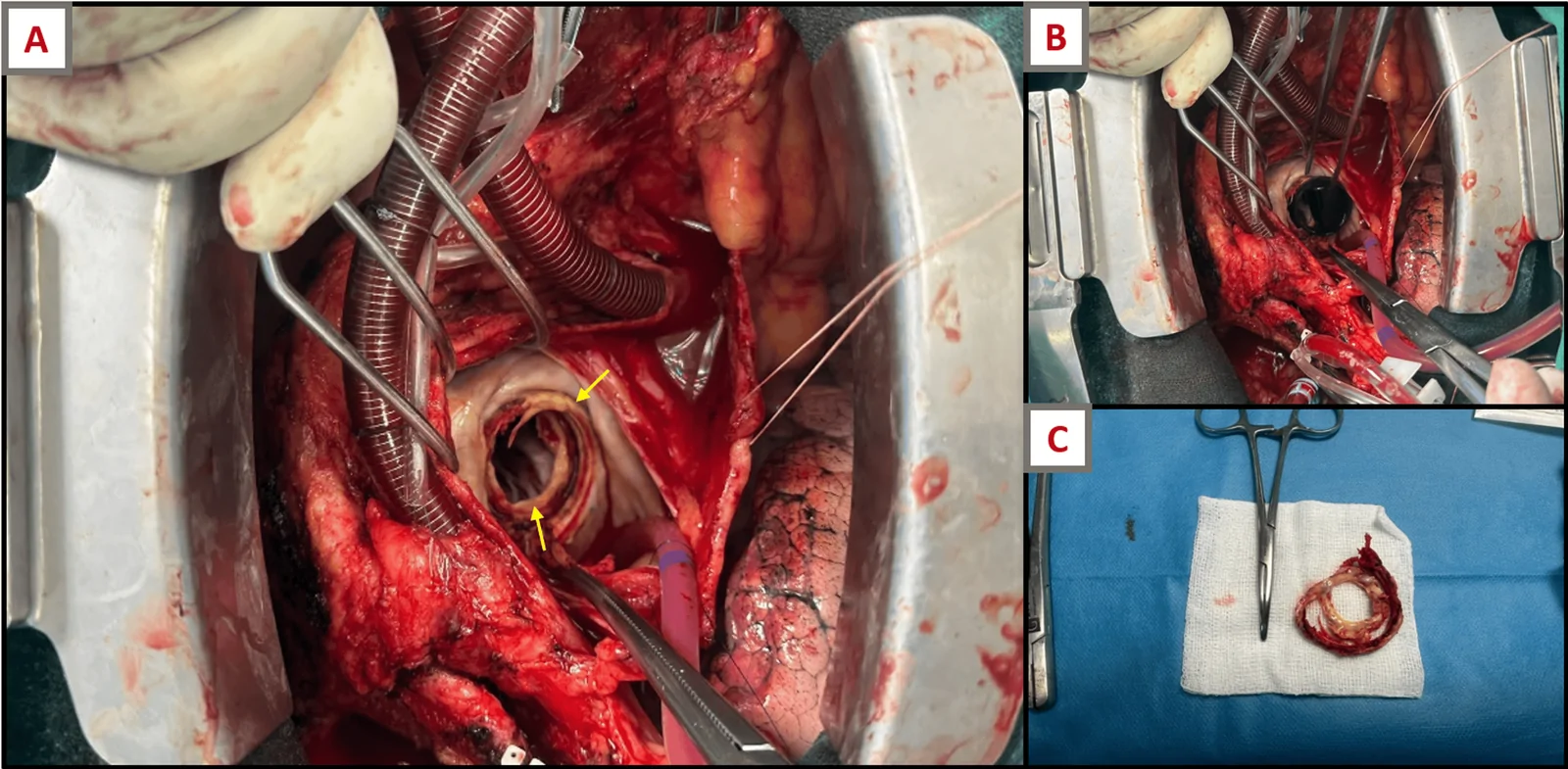
Intraoperative images of mitral pannus removal. (A) The mechanical mitral prosthesis was covered by a huge pannus surrounding the prosthetic ring (yellow arrow). (B) The mechanical mitral valve is clearly visible after removal of the ventricular-side pannus. (C) Gross appearance of the pannus tissue removed from the ventricular surface of the prosthesis.

The patient was referred to the cardiovascular surgery department for further management. Under extracorporeal circulation, stenosing subvalvular masses were surgically excised. Perioperative examination revealed dense fibrous tissue overgrowth surrounding the prosthetic rings and extending toward the leaflets, with a hard, whitish fibroconnective appearance characteristic of pannus formation, leading to leaflet immobilization and prosthetic obstruction (Figure 8).

Given the severity of prosthetic dysfunction, both the mitral and aortic prostheses were replaced. The definitive diagnosis was subsequently confirmed by histopathological examination.

Follow-up and outcomes

The postoperative course was favorable. At two-month follow-up, the patient reported complete resolution of chest pain and significant improvement in exercise tolerance. Control TTE demonstrated a marked reduction in transprosthetic gradients, with no postoperative complications or recurrent symptoms observed during follow-up.

Patient perspective

The patient reported a significant improvement in quality of life after surgery, particularly regarding dyspnea, exercise tolerance, and daily physical activities.

## Discussion

Although advances in prosthetic valve design and surgical techniques have significantly improved long-term outcomes, prosthetic valve dysfunction remains an important clinical challenge. Mechanical valve obstruction may result from several mechanisms, including thrombus formation, pannus overgrowth, infective endocarditis, or structural valve abnormalities [5]. The simultaneous occurrence of pannus involving both aortic and mitral mechanical prostheses is extremely uncommon. Most reports in the literature describe isolated involvement of a single prosthetic valve, either in the aortic or mitral position, making the present case a particularly unusual presentation [6,7].

Distinguishing pannus from thrombus is of major clinical importance because treatment strategies differ considerably. While prosthetic valve thrombosis may be managed with intensified anticoagulation or thrombolytic therapy in selected cases, pannus represents a fibrous tissue proliferation originating from the prosthetic sewing ring that progressively restricts leaflet mobility and generally requires surgical intervention [8]. The mechanisms underlying pannus development are not fully understood. Several factors have been implicated, including chronic inflammatory reactions to prosthetic material, abnormal local hemodynamic forces, prosthesis-related characteristics, and host-specific biological responses. Female sex and smaller prosthetic valve sizes have also been associated with an increased risk of pannus formation [9].

Pannus formation is an uncommon but clinically relevant late complication of prosthetic valve replacement. Sakamoto et al. reported pannus-related aortic prosthetic valve obstruction requiring reoperation in 1.8% of patients (7/390) [1]. Pannus may occur in both mechanical and bioprosthetic valves; however, in mechanical prostheses, fibrous tissue overgrowth may progressively restrict leaflet or disc motion and cause obstruction, whereas bioprosthetic valve dysfunction may additionally result from structural valve degeneration, including leaflet fibrosis and calcification [1,3]. Pannus is reported more frequently in the aortic position than in the mitral position. Progressive fibrous tissue growth may gradually encroach upon the prosthetic orifice, resulting in restricted leaflet excursion and increasing transprosthetic gradients. In some patients, pannus and thrombus may coexist, further complicating the diagnostic evaluation of prosthetic valve obstruction [10,11].

Because pannus develops slowly over time, diagnosis is often delayed until significant hemodynamic impairment occurs. Clinical manifestations are usually nonspecific and may include progressive dyspnea, reduced exercise capacity, or symptoms of heart failure. Echocardiography often demonstrates elevated gradients but may have limited ability to directly visualize pannus because of acoustic shadowing generated by the prosthetic material [12]. TTE remains the cornerstone of the initial assessment of prosthetic valve function by providing information on transprosthetic gradients, flow velocities, and valve hemodynamics. TEE offers improved visualization of prosthetic structures but can still be affected by imaging artifacts. Echocardiographic features suggestive of pannus include a small, dense, immobile echogenic lesion in a patient with a chronic clinical course and adequate anticoagulation, whereas a thrombus is generally larger and more mobile [12,13].

Cinefluoroscopy provides a direct assessment of prosthetic leaflet motion and is particularly useful for detecting abnormal opening or closing angles. Nevertheless, its ability to identify the nature of the obstructive lesion remains limited, and additional imaging is usually required to distinguish pannus from thrombus [8]. Cardiac CT has emerged as a valuable complementary imaging technique in the evaluation of prosthetic valve dysfunction. Its high spatial resolution allows detailed visualization of periprosthetic structures and improves the detection of subvalvular fibrous tissue that may not be adequately identified by echocardiography [12,14].

In addition, CT attenuation measurements contribute substantially to the differential diagnosis. Thrombotic material usually demonstrates low attenuation values, whereas pannus exhibits higher attenuation because of its fibrotic composition. Previous studies have shown that attenuation values above 145 HU strongly favor the diagnosis of pannus [15,16]. Cardiac CT has also been reported to outperform TEE in detecting pannus involving mitral mechanical prostheses [14]. Several CT-derived parameters have been correlated with the severity of prosthetic valve obstruction. Increased pannus thickness, greater encroachment of the prosthetic orifice, and larger pannus-to-valve diameter ratios have all been associated with more severe hemodynamic impairment and reduced leaflet mobility [17,18].

Unlike thrombotic obstruction, pannus does not regress with anticoagulation therapy. Therefore, surgery remains the treatment of choice in patients with clinically significant obstruction. Depending on intraoperative findings, management may involve isolated pannus excision or complete prosthetic valve replacement [9,19]. This case illustrates the diagnostic value of combining echocardiography, cinefluoroscopy, and cardiac CT in the evaluation of prosthetic valve dysfunction. The exceptionally rare involvement of both aortic and mitral mechanical prostheses by pannus highlights the importance of considering this diagnosis in patients presenting with progressive prosthetic obstruction despite adequate anticoagulation. Early recognition and prompt surgical management are essential to optimize clinical outcomes.

## Conclusions

Pannus formation is an uncommon but clinically significant cause of mechanical prosthetic valve obstruction and should be considered in patients presenting with progressive prosthetic dysfunction despite adequate anticoagulation. Multimodality imaging, particularly echocardiography, cinefluoroscopy, and cardiac CT, plays a pivotal role in establishing the diagnosis and differentiating pannus from thrombus. This case illustrates the exceptionally rare occurrence of simultaneous pannus-related dysfunction of both aortic and mitral mechanical prostheses and highlights the importance of early recognition and timely surgical management.
